# A Plea for Our Voluntary Hospitals

**Published:** 1888-12-29

**Authors:** 


### A Plea for Our Voluntary Hospitals.

The following letter appeared in the *Pall Mall Gazette* of 20th inst., and relates to the discussion in its columns of "Hospital Extravagance and Expenditure":—

"As the Christmas offerings are in many cases a matter of supreme importance to the maintenance and efficiency of some of our noblest and best hospitals, which are mainly supported by voluntary contributions, may I be allowed to enter a protest against the manner in which Mr. Michelli's points have been presented to your readers? 'Popular in form, but very misleading to the general reader,' would be a fair criticism of the articles in question. We must have hospitals; they need all the offerings of the charitable, and anything which tends to undermine public confidence in them, without good cause or necessity, is to be strongly deprecated. It must not be forgotten, and I can speak from actual knowledge, after a very careful personal inspection, that when everything has been said that can fairly be urged in criticism of our hospital system as a whole, our hospitals must be held to be far and away the best administered and most efficient in the world. Let the charitable therefore take heart this Christmas, and, having ascertained that the hospital of their choice has issued an annual report for say seven consecutive years, let them give to it freely and with both hands.

"It is of course difficult to summarise a long paper so as to faithfully reproduce the salient features without giving undue or increased force to much that it contains. The great drawback to Mr. Michelli's paper is its non-recognition of the willingness invariably displayed by the authorities of all well-managed hospitals throughout the country to give the fullest information to any one who displays a genuine interest or special knowledge, or who can show good cause for his troubling them. Another drawback is the necessary omission of the names of the hospitals specially quoted, but this will be removed by the publication next month of a full analysis of the accounts of the chief hospitals throughout the country, till which time I would urge your readers, in all fairness, to suspend judgment. Finally, Mr. Ryan, of St. Mary's Hospital, has done good service by proving from actual figures, what the initiated knew already, that the principal metropolitan and provincial hospitals can show a clean bill of health on almost, if not quite, all the chief points raised by the discussion. As Mr. Ryan points out, the effect of the first article you published was to mislead the reader, because it took no account of the discussion which followed, a very material omission, which, however unintentional, is calculated, as you will see from the enclosed report, 'to create a false impression in the public mind to the great damage of the numerous ably and economically managed London hospitals,' which, be it always remembered, comprise by far the greater portion of such institutions. If this had not been the case, I would not have troubled you with this letter.—I am, Sir, &c.,

"The Lodge, W., Dec. 18. Henry C. Burdett."

